# Association of serum intestinal fatty-acid binding protein, inflammatory and oxidative stress markers with colorectal cancer among Ghanaian patients

**DOI:** 10.1371/journal.pone.0333799

**Published:** 2025-10-03

**Authors:** Genevieve Kwao-Zigah, Antoinette Bediako-Bowan, Gabriel Atampugre Atampugbire, Caleb Koranteng Kwayisi-Darkwah, Osbourne Quaye, Gloria Kezia Aryee, Emmanuel Ayitey Tagoe

**Affiliations:** 1 Department of Biochemistry, Cell and Molecular Biology, West African Centre for Cell Biology of Infectious Pathogens (WACCBIP), School of Biological Sciences, University of Ghana, Accra, Ghana; 2 Department of Surgery, University of Ghana Medical School, University of Ghana, Korle-Bu, Accra, Ghana; 3 Department of Surgery, Korle Bu Teaching Hospital, Korle-Bu, Accra, Ghana; 4 Department of Medical Laboratory Sciences, School of Biomedical and Allied Health Sciences, University of Ghana, Korle-Bu, Accra, Ghana; Tabriz University of Medical Sciences, IRAN, ISLAMIC REPUBLIC OF

## Abstract

**Background:**

In sub-Saharan Africa, colorectal cancer (CRC) is becoming an increasingly serious public health issue. However, little is known about the relationships between inflammation, oxidative stress, and intestinal barrier biomarkers in African populations. This study examined the association between selected biomarkers, dietary factors, and the risk of CRC among treatment-naïve patients in Ghana.

**Methods:**

This hospital-based analytical cross-sectional study included twenty-eight CRC patients and twenty-six non-CRC volunteers. Serum samples processed from whole blood were analyzed for tumor necrosis factor-α (TNF-α), interleukin-1β (IL-1β), and intestinal fatty acid binding protein (iFABP) levels using enzyme-linked immunosorbent assays (ELISA). The thiobarbituric acid test was used to measure the concentration of serum malondialdehyde (MDA). Dietary intake data were collected using structured questionnaires. Logistic regression models were used to evaluate associations with CRC risk.

**Results:**

CRC patients showed significantly higher levels of TNF-α, IL-1β, MDA and iFABP compared to the control group (p < 0.05). A Pearson correlation analysis revealed a significant positive correlation between IL-1β and TNF-α levels (r = 0.606, p < 0.000). TNF-α and MDA levels were significantly associated with increased odds of CRC in both crude and adjusted models (p < 0.05). In contrast, IL-1β and iFABP showed only crude significant associations with CRC. Dietary factors, including alcohol consumption, red/processed meat, vegetables, and dairy intake, were not significantly associated with CRC risk.

**Conclusion:**

The study identifies TNF-α and MDA as potential biomarkers associated with CRC, emphasizing the role of inflammation and oxidative stress in CRC pathogenesis in Ghana. The lack of adjusted association with iFABP may reflect population-specific patterns or limited statistical power. Further large-scale longitudinal studies are warranted.

## Introduction

Colorectal cancer (CRC) is a major cause of cancer-related morbidity and mortality globally, ranking third in incidence and second in mortality [1]. While the majority of CRC cases have historically occurred in countries with a high Human Development Index (HDI) [2], there has been a rapid rise in incidence in low- and middle-income countries, including those in sub-Saharan Africa [3]. This rise has been linked to increasing adoption of “Westernized” diets and lifestyles, as well as environmental exposures and genetic predisposition [1]. However, limited information exists on how biological and lifestyle risk factors interact to drive CRC development in many African countries, including Ghana.

Chronic inflammation, often triggered by dietary irritants, gut dysbiosis, or recurring infections, creates a pro-tumorigenic microenvironment characterized by the release of pro-inflammatory cytokines, including interleukin-1 beta (IL-1β), interleukin-6 (IL-6), and tumor necrosis factor-alpha (TNF-α) [4]. These cytokines can promote angiogenesis, inhibit apoptosis, and enhance cellular proliferation, all of which contribute to the development of colorectal cancer. Concurrently, inflammation triggers the production of reactive oxygen species, resulting in oxidative stress [5]. These reactive molecules can damage DNA, induce lipid peroxidation, and modify proteins, leading to genomic instability and alterations in tumor suppressor genes or oncogenes [6]. Malondialdehyde (MDA), a byproduct of lipid peroxidation and a key biomarker of oxidative stress, has been associated with increased CRC risk [7]. Collectively, the inflammatory-oxidative axis forms a feedback loop that amplifies inflammatory signaling pathways such as NF-κB and STAT3, which are frequently activated in CRC [4].

Intestinal barrier disruption, commonly referred to as “leaky gut,” has been increasingly implicated in CRC pathogenesis. The translocation of microbial products via the leaky gut activates pro-inflammatory pathways, including NF-κB and STAT3, which contribute to epithelial injury, DNA damage, and tumorigenesis [8]. Intestinal fatty acid-binding protein (iFABP) is a recognized marker of intestinal epithelial injury, with elevated levels observed in conditions associated with gut barrier dysfunction [9].

One of the most controllable risk factors for CRC is diet. Consumption of processed and red meat has been consistently associated with an increased risk of CRC [10,11]. Conversely, a lower risk of CRC has been linked to increased consumption of vegetables and dairy products [12,13]. Alcohol consumption is another risk factor, contributing to CRC development through mechanisms such as oxidative stress and DNA damage mediated by acetaldehyde [14]. The majority of this evidence, however, stems from research conducted in high-income nations, and it remains unclear whether these associations hold in African populations, where dietary habits and food preparation methods differ significantly.

The study aimed to evaluate the associations between inflammatory (TNF-α, IL-1β), oxidative stress (MDA), and intestinal barrier dysfunction (iFABP) biomarkers, along with key dietary factors, alcohol, red/processed meat, vegetable, and dairy intake, and the risk of CRC among treatment-naïve patients in Ghana.

## Materials and methods

### Study design and population

The study was a hospital-based analytical cross-sectional study conducted at the Korle-Bu Teaching Hospital-Ghana from February to September 2023. The sample size was calculated for a finite population of 150 (the average number of surgeries performed by the KBTH surgical colorectal unit in a year), using a 95% confidence interval, 5% margin of error, and 50% population proportion, giving an initial sample size of 109. The finite population correction was applied to the sample size (109); the n-adjusted sample size was 63.

The design used was an analytical cross-sectional approach to recruit 28 patients with histologically confirmed colorectal cancer and 26 healthy individuals as controls. Control participants were individuals examined at the Colonoscopy Unit who were confirmed to be CRC-free, and patients include those with newly diagnosed CRC after colorectal examination or already diagnosed with CRC patients who have been referred to the facility. Inclusion criteria were patients who had not yet received any neoadjuvant therapy (such as chemotherapy or radiotherapy), had no personal history of any other type of cancer, had no known family history of colorectal cancer, and had no diagnosis of inflammatory bowel disease, no antibiotic use within the three months before the study, and chronic infections such as human immunodeficiency virus (HIV), tuberculosis and other chronic diseases. Body weight was measured using a calibrated digital scale with participants wearing light clothing and no shoes. Height was measured using a stadiometer with participants standing upright against a wall without shoes. Body Mass Index (BMI) was then calculated as weight in kilograms divided by height in meters squared (kg/m^2^).

Informed consent was obtained from all study participants. The study was approved by the Korle-Bu Teaching Hospital Institutional Review Board (KBTH-STC/IRB/000130/2022) and the Ethics Committee of Basic and Applied Sciences (ECBAS), University of Ghana (ECBAS 052/21–22).

### Blood sample collection

Five milliliters (5) ml of venous blood were collected from each participant using a serum-separator vacutainer. Following centrifugation, the serum was separated, transferred into labeled Eppendorf tubes, and stored at −80 °C for subsequent analysis.

### Biochemical analysis

Serum levels of iFABP, MDA, TNF-α, and IL-1β were measured in the study participants. iFABP, TNF-α, and IL-1β levels were determined using commercilally available human sandwich enzyme-linked immunosorbent assay (ELISA) kit (SunLong Biotech Co.,Ltd, China), following the manufacturer’s protocol. Concentrations were expressed in pg/mL. The assay sensitivity was 5.5 pg/mL, with a measurement range of 15.6 pg/mL – 1000 pg/mL. MDA levels were assessed using the thiobarbituric acid reactive substance (TBARS) test. Absorbance of each sample was read at 532nm, and calibration curve prepared with triethoxypropane (TEP) standards. Absorbance readings were taken for the ELISA kits at 450 nm. Calibrants provided in the respective kits were used to generate standard curves for the quantification of iFABP, TNFα and Ilβ levels. All absorbance readings were recorded with a microplate reader (Thermo Scientific VARIOSKAN LUX). 

### Statistical analysis

Statistical analysis was conducted using the IBM Statistical Package for the Social Sciences (SPSS) version 25 analysis software. Continuous values were expressed as mean ± standard deviation (SD), while categorical data were presented as frequencies and percentages. Normality was assessed using the Shapiro-Wilk test, and since the data were not normally distributed, the non-parametric Mann–Whitney U test was used to compare continuous variables between the CRC and control groups. The Fisher’s exact test was used to assess differences in proportions between groups for categorical variables.

The Pearson correlation was used to determine the relationship between TNFα and serum markers (IL-1β, MDA, and iFABP levels). Univariate and multivariate logistic regression were used to determine the association between CRC and serum markers (TNFα, IL-1β, MDA, iFABP) as well as dietary factors. A *p*-value < 0.05 was considered statistically significant.

## Results

### Clinicopathological and dietary characteristics of study participants

The clinicopathological and dietary characteristics of the study participants are summarized in Table 1. No significant differences in baseline characteristics were observed between the case and control groups. The mean age of participants in the CRC group was 53 years, compared to 48 years in the control group. Among the CRC cases, 15 (53.6%) were male and 13 (46.4%) were female, resulting in a male-to-female ratio of 1.2:1. Half (50.0%) of CRC tumors were located at the rectum, with 64.3% (18/28) at histological grade 2 and 60.7% (17/28) at stage 2. Dietary characteristics were largely similar between the two groups, with no significant differences in the consumption of red/processed meat (p > 0.05), vegetables/fruits (p > 0.05), or dairy products (p > 0.05). Alcohol consumption also did not differ significantly, 35.7% (10/28) of cases and 34.6% (9/26) of controls reporting alcohol use (p > 0.05).

**Table 1:** Baseline and dietary characteristics of study participants.

Characteristics	CRC patients (N = 28)	Control group (N = 26)	p-value
Age (Mean ± SD) (yrs)	53.32 ± 14.6	47.5 ± 16.9	0.178
BMI (mean ± SD) (kg/m^2^)	25.28 ± 3.77	27.52 ± 6.23	0.122
*Sex: n (%)*			
Male	15 (53.6)	15 (57.7)	0.791
Female	13 (46.4)	11 (42.3)	
*Location of tumor: n (%)*			
Right colon	7 (25.0)		
Left colon	7 (25.0)	–	–
Rectum	14 (50.0)		
*Grade: n (%)*			
Well-differentiated	5 (17.9)		
Moderately differentiated	18 (64.3)	–	–
Poorly differentiated	5 (17.9)		
*Stage: n (%)*			
I	8 (28.6)		
II	17 (60.7)	–	–
III	3 (10.7)		
*Alcohol consumption: n (%)*			
No	6 (21.4)	12 (46.2)	0.088
Yes	10 (35.7)	9 (34.6)	
Used to	12 (42.9)	5 (19.2)	
*Red/processed meat: n (%)*			
1-3 times a month	5 (17.9)	7 (26.9)	0.725
1-3 times a week	11 (39.3)	7 (26.9)	
Most meals	7 (25.0)	6 (23.1)	
Used to	5 (17.9)	6 (23.1)	
*Vegetable/fruit intake: n (%)*			
1-3 times a month	13 (46.4)	8 (30.8)	0.498
1-3 times a week	9 (32.1)	11 (42.3)	
Most meals	6 (21.4)	7 (26.9)	
*Dairy products: n (%)*			
Used to	5 (17.9)	7 (26.9)	0.109
1-3 times a month	13 (46.4)	4 (15.4)	
1-3 times a week	5 (17.9)	7 (26.9)	
Most meals	5 (17.9)	8 (30.8)	

N = population. Categorical data is presented as frequency (percentage), and continuous data is mean ± standard deviation. Fisher’s exact test was used to test for proportion difference, and Mann-Whitney U test for mean difference. p-value < 0.05 is considered statistically significant.

### Biochemical analysis of serum markers in study participants

**Fig 1:**
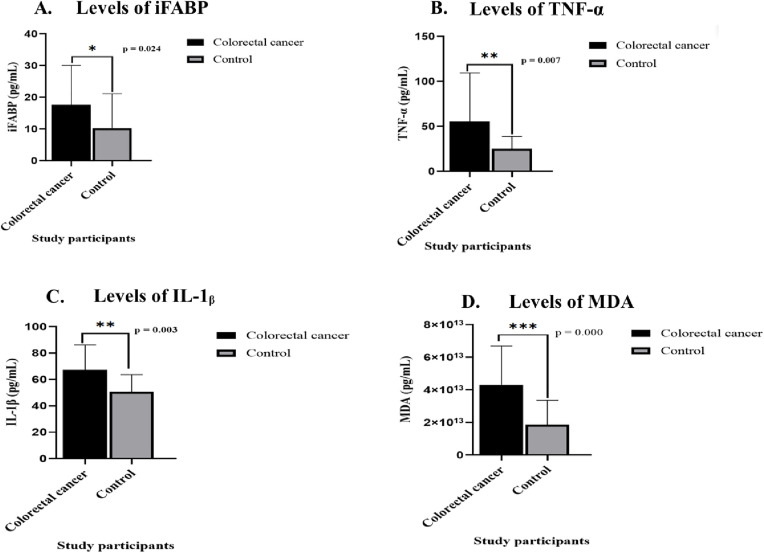
Levels of iFABP, TNF-α, IL-1β, and MDA in CRC and control group. A significantly higher level of iFABP, TNF-α, IL-1β, and MDA in CRC patients compared to the control group. (A) iFABP = intestinal fatty-acid binding protein, (B) TNF-α = tumor necrosis factor-α, (C) IL-1β = interleukin-1β and (D) MDA = malondialdehyde. * p ≤ 0.05, ** p ≤ 0.01, and p ≤ 0.001.

Levels of iFABP, TNF-α, IL-1β, and MDA in patients with CRC compared to controls are shown in Fig 1.

The mean levels of iFABP were significantly higher in CRC patients than in the control group (p < 0.024). The levels of inflammatory cytokines, including TNF-α and IL-1β, were significantly elevated in patients with CRC compared to controls. Levels of MDA were significantly elevated in the CRC patients as compared to controls (p < 0.001). The raw data used to generate Fig 1 and all analysis is provided in S1 File.

### Correlation of TNF-α levels with serum indicators

The correlation between TNF-α levels and serum markers is presented in Table 2. A positive and significantly strong correlation was observed between study participants’ TNF-α levels and IL-1β levels (r = 0.606, p > 0.001). All other indicators were not significantly correlated with TNF-α levels (p > 0.05).

**Table 2:** Correlation between TNF-α levels and serum markers in study participants.

Variables	Pearson correlation coeff.. (r)	p-value
IL-1β	0.606	0.000*
MDA	0.191	0.204
iFABP	−0.081	0.581

r- Pearson’s correlation coefficient; *p-value <0.05 were considered statistically significant.

### Association of CRC with predictor variables

Univariate and multivariate logistic regression analyses are used to establish a relationship between intestinal permeability, inflammation, oxidative stress, nutritional status, and CRC, as summarized in Table 3. iFABP, TNF-α, and MDA were associated with CRC (p < 0.05). After adjusting for confounders, BMI (aOR = 1.26, 95% CI = 1.05–1.50, p < 0.05), TNF-α (aOR = 1.09, 95% CI = 1.02–1.16, p < 0.01), and MDA (aOR = 1.06, 95% CI = 1.02–1.11, p < 0.01) were independent correlates for CRC.

**Table 3:** Univariate and multivariate logistic regression of serum markers and dietary indicators with CRC.

Variable	Unadjusted Odds Ratio		Adjusted Odds Ratio	
	OR (95% CI)	p-value	aOR (95% CI)	p-value
*Age*	1.03 (0.99–1.06)	0.177	1.05 (1.00–1.10)	0.070
*BMI (mean ± SD) (Kg/m*^*2*^)	1.10 (0.98–1.23)	0.122	1.26 (1.05–1.50)	0.012*
*Sex: n (%)*				
Male	1	–	1	–
Female	0.85 (0.29–2.48)	0.761	0.51 (0.12–2.25)	0.373
*Alcohol consumption*				
No	1	–	1	–
Yes	4.80 (1.15–20.09)	0.032*	5.53 (0.93–32.87)	0.060
Used to	2.16 (0.54–8.57)	0.273	1.40 (0.22–8.93)	0.726
*Red/processed meat*				
Used to	1	–	1	–
1-3 times a month	0.86 (0.16–4.47)	0.855	1.09 (0.14–8.59)	0.937
1-3 times a week	1.87 (0.41–8.61)	0.413	4.71 (0.61–36.42)	0.137
Most meals	1.40 (0.28–7.02)	0.682	5.97 (0.62–57.83)	0.123
*Vegetable/fruit intake*				
1-3 times a month	1	–	1	–
1-3 times a week	0.50 (0.15–1.75)	0.280	0.99 (0.15–6.45)	0.988
Most meals	0.53 (0.13–2.14)	0.371	0.37 (0.08–1.65)	0.192
*Dairy products*				
Used to	1	–	1	–
1-3 times a month	4.55 (0.92–22.63)	0.064	5.15 (0.56–47.63)	0.149
1-3 times a week	1.00 (0.20–5.07)	1.000	0.22 (0.01–3.36)	0.274
Most meals	0.88 (0.18–4.34)	0.870	0.58 (0.04–8.36)	0.688
iFABP	1.06 (1.00–1.12)	0.035*	1.05 (0.99–1.12)	0.090
TNF-α	1.07 (1.02–1.12)	0.008*	1.09 (1.02–1.16)	0.009*
IL-1β	1.09 (1.02–1.17)	0.011*	1.07 (0.991.16)	0.083
MDA	1.06 (1.02–1.10)	0.002*	1.06 (1.02–1.11)	0.005*

OR- crude odds ratio; aOR- adjusted odds ratio; 95% CI- 95% confidence interval;. iFABP = intestinal fatty-acid binding protein, TNF-α = tumor necrosis factor-α, IL-1β = interleukin-1β and MDA = malondialdehyde. *p < 0.05 is considered statistically significant.

## Discussions

Chronic inflammation, oxidative stress, and intestinal barrier dysfunction are critical contributors to colorectal cancer (CRC) development. These processes interact to create a pro-tumorigenic microenvironment characterized by immune dysregulation, oxidative stress, and microbial translocation. In this study, we investigated the relationships among key inflammatory and intestinal barrier-related biomarkers to better understand their interconnection in CRC. Our findings revealed that CRC patients had significantly higher serum levels of iFABP, TNF-α, IL-1β, and MDA compared to healthy controls. These results suggest a strong association between intestinal barrier dysfunction, chronic inflammation, and oxidative stress in colorectal carcinogenesis.

TNF-α and IL-1β are pro-inflammatory cytokines that play central roles in mediating inflammatory responses. During the early stages of CRC, there is often an overexpression of inflammatory signaling molecules, including TNF-α, interleukin-1β (IL-1β), and IL-4. In our study, CRC patients showed significantly higher serum levels of TNF-α and IL-1β compared to controls, consistent with findings from previous studies conducted in Brazil [15], Poland [16], and Romania [17], which also reported elevated levels of these cytokines in CRC patients. Overexpression of TNF-α mRNA and IL-1β has been associated with increased tumor growth and invasion, both in epithelial and metastatic CRC [18]. In our analysis, IL-1β was positively and significantly correlated with TNF-α, suggesting a synergistic interaction in inflammatory signaling pathways. TNF-α is known to stimulate the production of IL-1β, and vice versa, primarily through the activation of nuclear factor-kappa B (NF-κB) and other transcription factors involved in chronic inflammation [19]. This feedback loop enhances the inflammatory milieu, potentially contributing to tumor initiation, angiogenesis, and immune evasion. However, the association of IL-1β with CRC risk was not statistically significant after adjusting for confounders in multivariate logistic regression.

This result may indicate the impact of specific risk factors, including age, BMI, or lifestyle habits, which can increase IL-1β levels independently of CRC. Moreover, IL-1β is strongly associated with other pro-inflammatory agents like TNF-α, sharing common signaling pathways. As a result, the presence of collinearity with more dominant predictors could diminish its standalone predictive significance [19]. Also, the relatively small sample size in this study might have constrained the statistical power of the multivariate model, resulting in the loss of significance for IL-1β after adjustment. Notably, elevated serum TNF-α levels were significantly associated with increased odds of CRC in both crude and adjusted logistic regression models, underscoring the strong and independent link between systemic inflammation and CRC risk [20]. These findings further support the potential use of TNF-α as a biomarker for CRC risk assessment.

Elevated levels of malondialdehyde (MDA), a byproduct of lipid peroxidation, reflect increased oxidative stress and have been associated with poorer survival outcomes in CRC patients. In this study, CRC cases showed significantly higher serum MDA levels compared to controls, consistent with findings from previous studies in Iraq [7] and Poland [21,22], which also reported elevated MDA levels in CRC patients. MDA can react with nucleophilic sites on DNA to form adducts such as MDA-deoxyguanosine, which can lead to mutations [23]. These mutations in tumor suppressors or oncogenes may contribute to cancer development, including CRC. Furthermore, MDA generated through oxidative stress can activate NF-κB and other transcription factors, stimulating the production of pro-inflammatory cytokines, including TNF-α, IL-1, and IL-6. This creates an inflammatory environment that is favorable to tumor promotion and progression. In both crude and adjusted logistic regression analyses, elevated MDA levels were significantly associated with increased odds of CRC, highlighting oxidative stress as a major and independent factor in the pathophysiology of CRC. This finding aligns with earlier research from Bosnia, which identified MDA as an independent risk factor for CRC metastasis [24]. Overall, our results support the notion that oxidative damage actively contributes to DNA mutations, genomic instability, and malignant transformation [6] (all of which are characteristics of cancer), rather than being merely a byproduct of tumor presence.

Elevated iFABP levels in CRC patients suggest increased intestinal barrier dysfunction or intestinal permeability. Data on iFABP levels in CRC patients who have not undergone neo-adjuvant therapy remain limited. However, our findings are consistent with previous studies conducted in the Netherlands [25], Egypt [26], and South Africa [27], which have reported elevated iFABP levels in individuals with compromised intestinal barrier function. iFABP is considered a sensitive marker of mucosal integrity loss, released into the bloodstream upon enterocyte damage [28]. Disruption of the gut barrier may facilitate the translocation of toxins and microbial products, which could lead to chronic inflammation and CRC development. In our study, iFABP was significantly associated with CRC in univariate logistic regression analysis, indicating that individuals with elevated serum iFABP levels had higher odds of having CRC.

However, this association lost statistical significance in the multivariate model after adjusting for potential confounders. This attenuation suggests that the initial association observed in the crude model may have been confounded or mediated by variables such as age, BMI, diet, or systemic inflammation. The lack of significance in the adjusted model may also indicate potential collinearity between iFABP and other inflammatory or oxidative stress markers. Additionally, the small sample size may have hampered the statistical power, contributing to the loss of significance.

Our study supports previously published findings that indicate increased BMI is associated with higher odds of developing CRC [29,30]. Several mechanisms have been proposed to explain this association, including insulin resistance, chronic low-grade inflammation, and altered levels of adipokines in individuals with higher BMI, all of which contribute to a pro-tumorigenic milieu in the colon [31]. Additionally, obesity has been linked to changes in gut microbiota composition, increased oxidative stress, and impaired immune surveillance, further facilitating the development and progression of CRC [32].

In contrast, our study did not observe significant associations between dietary factors and CRC risk. This finding diverges from previous studies that reported significant associations between CRC and alcohol consumption [33,34], red and processed meat [35,36], vegetables and fruits [12,37], and dairy products [13]. A potential explanation for this discrepancy may lie in the regional differences in dietary habits, food types, and preparation methods. Specifically, the dietary patterns prevalent in Ghana may differ substantially from those in Western populations, potentially accounting for the absence of associations typically observed in other settings.

### Strengths and limitations

To adopt a comprehensive, multidimensional approach to CRC risk assessment, the current study evaluated biological markers associated with inflammation, oxidative stress, and intestinal barrier dysfunction, alongside dietary factors. One significant advantage of this study is its inclusion of only treatment-naive CRC patients, thereby minimizing potential confounding effects from neoadjuvant therapy on lifestyle exposures and biomarker profiles. Conducting the study in Ghana also contributes relevant region-specific data to the global literature, addressing a critical research gap in sub-Saharan Africa and supporting the development of context-appropriate public health strategies. Furthermore, the reporting of non-significant associations, particularly for dietary factors, provides an important contribution to the literature by emphasizing the complexity of diet–disease relationships in diverse populations.

However, the relatively small sample size limits statistical power and may account for the observed non-significant associations, especially with dietary variables. The cross-sectional design precludes causal inference, and reliance on self-reported dietary data introduces recall bias. Biomarkers assessed at a single time point may not accurately reflect chronic exposures, and unmeasured confounders such as genetic predisposition, lifestyle, and comorbidities may have influenced the findings. Furthermore, the lack of intake stratification and the diversity of local dietary patterns may have obscured potential dose–response relationships.

## Conclusion

Our study reports a significantly elevated levels of iFABP, TNF-β, IL-1β and MDA in CRC patients than in controls. This study also suggests that oxidative stress and inflammation, particularly elevated MDA and TNF-α levels, may play a significant role in the pathophysiology of colorectal cancer among untreated individuals. This study also provides useful region-specific data and emphasizes the importance of further investigating in intestinal permeability, oxidative and inflammatory pathways in CRC. To validate these findings, a larger prospective studies incorporating more thorough dietary and biomarker profiling are necessary.

## Supporting information

S1 File The raw data used to generate Fig. 1 and all analysis. (XLSX)
